# Pretreatment Prediction of Relapse Risk in Patients with Osteosarcoma Using Radiomics Nomogram Based on CT: A Retrospective Multicenter Study

**DOI:** 10.1155/2021/6674471

**Published:** 2021-02-04

**Authors:** Jin Liu, Tao Lian, Haimei Chen, Xiaohong Wang, Xianyue Quan, Yu Deng, Juan Yao, Ming Lu, Qiang Ye, Qianjin Feng, Yinghua Zhao

**Affiliations:** ^1^Department of Radiology, The Third Affiliated Hospital of Southern Medical University, Guangzhou, Guangdong 510630, China; ^2^Guangdong Provincial Key Laboratory of Medical Image Processing, School of Biomedical Engineering, Southern Medical University, Guangzhou 510515, China; ^3^Department of Radiology, Third Affiliated Hospital of Sun Yat-sen University, Guangzhou, Guangdong 510630, China; ^4^Department of Radiology, Zhujiang Hospital of Southern Medical University, Guangzhou, Guangdong 510282, China; ^5^Department of Radiology, The First Affiliated Hospital of Guangzhou Medical University, Guangzhou, Guangdong 510120, China; ^6^Department of Pathology, The Third Affiliated Hospital of Southern Medical University, Guangzhou, Guangdong 510630, China; ^7^Department of Oncology, The Third Affiliated Hospital of Southern Medical University, Guangzhou, Guangdong 510630, China

## Abstract

**Objective:**

To develop and externally validate a CT-based radiomics nomogram for pretreatment prediction of relapse in osteosarcoma patients within one year.

**Materials and Methods:**

In this multicenter retrospective study, a total of 80 patients (training cohort: 63 patients from three hospitals; validation cohort: 17 patients from three other hospitals) with osteosarcoma, undergoing pretreatment CT between August 2010 and December 2018, were identified from multicenter databases. Radiomics features were extracted and selected from tumor regions on CT image, and then, the radiomics signature was constructed. The radiomics nomogram that incorporated the radiomics signature and clinical-based risk factors was developed to predict relapse risk with a multivariate Cox regression model using the training cohort and validated using the external validation cohort. The performance of the nomogram was assessed concerning discrimination, calibration, reclassification, and clinical usefulness.

**Results:**

Kaplan-Meier curves based on the radiomics signature showed a significant difference between the high-risk and the low-risk groups in both training and validation cohorts (*P* < 0.001 and *P* = 0.015, respectively). The radiomics nomogram achieved good discriminant results in the training cohort (*C*-index: 0.779) and the validation cohort (*C*-index: 0.710) as well as good calibration. Decision curve analysis revealed that the proposed model significantly improved the clinical benefit compared with the clinical-based nomogram (*P* < 0.001).

**Conclusions:**

This multicenter study demonstrates that a radiomics nomogram incorporated the radiomics signature and clinical-based risk factors can increase the predictive value of the osteosarcoma relapse risk, which supports the clinical application in different institutions.

## 1. Introduction

Osteosarcoma (OS) is the most common primary malignant bone tumor [1]. 30-40% of patients with localized osteosarcoma will subsequently develop relapse (including local recurrence and distant metastasis, resulting in a 5-year survival rate of only 23% to 29%) [2]. Approximately 60% of these patients will relapse in less than one year after surgery [3]. The study from the Rizzoli Orthopaedic Institute (Bologna, Italy) indicated that the relapse of osteosarcoma was closely related to the correct margin of surgery and the effectiveness of preoperative chemotherapy [4]. Previous documents have pointed out that the pivotal questions to be answered were lack of biomarkers, a wide tumor heterogeneity, and a rapid metastasizing potential [5]. So, seeking predictors of relapse within one year might be very important to benefit osteosarcoma patients from effective chemotherapy, additional surgery met the requirements, and supplementary radiotherapy [6]. However, it is still a complex challenge to accurately predict the relapse in osteosarcoma.

The probability of tumor relapse could be predicted using the Huvos grading system in osteosarcoma patients; however, this grading system is assessed only in the resected specimens after surgery [7]. Meanwhile, clinical risk factors (such as patient age, tumor stage, alkaline phosphatase vascular invasion, and joint invasion) were also used to predict relapse of osteosarcoma [8–11]. However, these clinical pathology factors could not reflect the heterogeneity of tumor, which is the main reason that leads to failure of prediction accuracy for the relapse in osteosarcoma [12, 13].

Currently, previous studies have shown that radiomics data contains strong prognostic information about cancers, such as osteosarcoma, lung cancer, and head-and-neck cancer, which are associated with potential gene expression patterns. Radiomics combined with clinical factors has improved the accuracy of survival prediction in cancer patients [14, 15]. Computed tomography (CT) scans can assess bone destruction and osteoid formation and have a greater advantage in assessing osteosarcoma. In addition, CT examination is a traditional, very conventional, low-cost inspection for patients [16]. Therefore, integrating clinical information into the radiomics model would be a promising direction for the prediction of relapse in osteosarcoma patients. However, regarding CT, research on the prediction of osteosarcoma relapse using this radiomics is relatively limited. Although CT radiomics analyses have recently been explored for evaluation of neoadjuvant chemotherapy response [17] and prediction survival in high-grade osteosarcoma [14], the results lacked essential multicenter external validation.

The purpose of this study was to predict the risk of relapse and to stratify the risks of relapse in patients with osteosarcoma within one-year postsurgery, using radiomics nomograms combined with radiomics features and clinical risk factors based on multicenter data.

## 2. Materials and Methods

### 2.1. Patients

**Figure 1:**
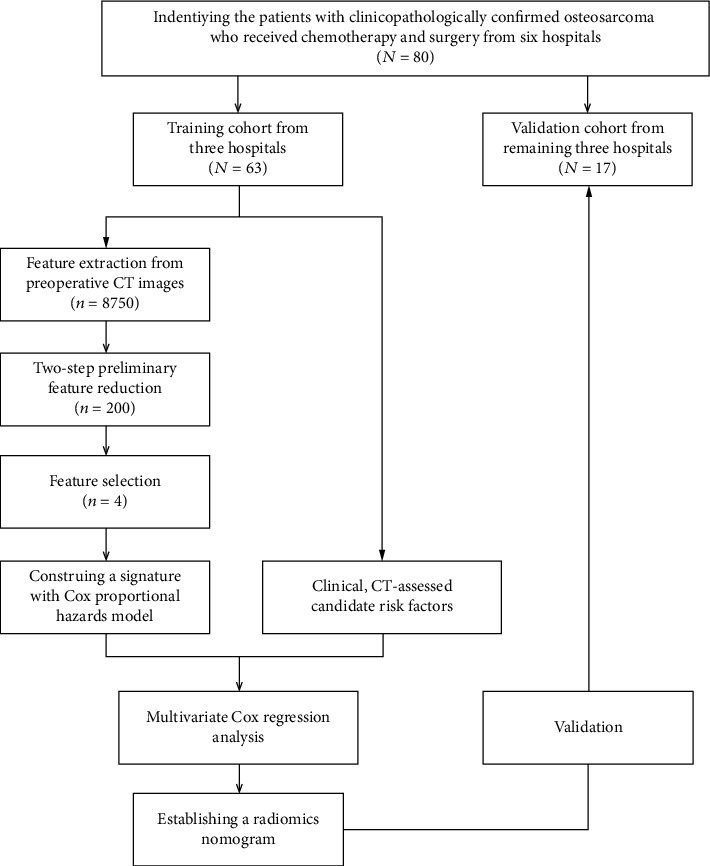
The work flow of this study.

This multicenter retrospective study was conducted by the main large-scale six hospitals in Guangdong Province and obtained ethical approval from the institutional review board (IRB) of the Third Affiliated Hospital of Southern Medical University, and the need for informed consent from the patients was waived. The entire design of this study is illustrated in Figure 1. The selection process of patients is illustrated in Supplementary Figure S1. According to the inclusion and exclusion criteria, a total of 80 osteosarcoma patients (49 males and 31 females; mean age, 25.59 ± 15.74 years; range, 5–71 years) with postoperative histopathology were retrospectively enrolled from multiple centers between August 2010 and December 2018. Treatments included preoperative neoadjuvant chemotherapy and surgery to achieve a wide excision. All patients received the treatments following the National Comprehensive Cancer Network (NCCN) guidelines (Supplementary 2) and were followed up in a timely fashion in the different hospitals (Supplementary 3) [18]. The patients with osteosarcoma who suffered a local early recurrence or distant metastasis within one year were defined as the relapse cohort, whereas the patients without relapse within one year were classified as the nonrelapse cohort. Supplementary 4 describes the criteria of serum markers (alkaline phosphatase and hemoglobin). Tumor-invading joints were determined as surface destruction and irregular edges in subarticular bone and overlying articular cartilage, joint space widened, or soft tissue mass in the close joint [19]. The differences in sex, age, HGB, ALP, pathological fracture, tumor location, morphology (including solidity, sphericity, and irregularity), and joint invasion between the two cohorts were assessed by using independent sample *t*-test or chi-squared test, where appropriate (*P* < 0.05).

### 2.2. Image Acquisition, Segmentation, and Feature Extraction

No preprocessing or normalization methods were used on the original images. The details regarding the CT acquisition parameters are shown in Table S1. ITK-SNAP software (http://www.itksnap.org) was used to obtain a region of interest (ROI) segmentation result. All ROI areas are delineated manually slice by slice of each osteosarcoma in transverse orientation around the gross tumor volume (GTV) on CT images without contrast-enhanced by doctors. The ROI-based radiomics features were extracted according to the methods described in Supplementary 5. All features conform to the definitions in the Image Biomarker Standardization Initiative (IBSI) [20]. The interobserver reproducibility and stability for feature extraction were initially analyzed with 40 randomly selected patients through ROI segmentation by two experienced radiologists, respectively (radiologists 1 and 2, with 20 and 12 years of experience in OS CT interpretation, respectively). The intraclass correlation coefficient (ICC) was used to assess the agreement of two radiologists in the CT characteristics. We interpreted an ICC of 0.80-1.00 as almost perfect agreement, 0.61-0.79 as substantial agreement, 0.41-0.60 as moderate agreement, 0.21-0.40 as fair agreement, and 0-0.20 as poor or no agreement. The segmentation for the remaining images was completed by radiologist 1.

### 2.3. Radiomics Feature Selection and Construction of the Radiomics Signature

Optimal feature reduction and selection were performed in two steps from the training cohort (Supplementary 6). According to the Harrell guideline, the number of events should exceed the number of included covariates by at least 10 times in a multivariate analysis [21]. Features with the almost perfect agreement remained for further screening. Features with low correlation with labels and highly redundant to each other were excluded using the Minimum Redundancy Maximum Relevance (MRMR) method [22]. The least absolute shrinkage and selection operator (LASSO) Cox regression model was used to select the most predictive features [23]. The selected features were then combined into a radiomics signature. Specifically, a radiomics score (Rad-score) was computed for each patient through a linear combination of the selected features weighted by their respective coefficients.

### 2.4. Validation of the Radiomics Signature

The potential association of radiomics signature and relapse risk was assessed in the training cohort and then validated in the validation cohort by using Kaplan-Meier survival analysis. The patients were classified into high-risk or low-risk groups according to the Rad-score; the threshold of which was identified by using X-tile [24]. The difference in the relapse curves of the high-risk and low-risk groups was evaluated by using a weighted log-rank test (the *G*-rho rank test, rho = 1) [25].

### 2.5. Construction and Assessment of the Radiomics Nomogram

To demonstrate the incremental value of the radiomics signature for an individualized assessment of relapse risk in patients with OS, both a radiomics nomogram and a clinical-based nomogram were constructed for the training cohort using the multivariate Cox analysis. The radiomics nomogram incorporated the radiomics signature and the clinical-based risk factors (including clinical and CT-accessed risk factors). The clinical-based nomogram contained only the clinical-based risk factors.

To compare the prediction performance of the radiomics nomogram, the performance was assessed in both the training and the validation cohorts with respect to discrimination, calibration, and clinical usefulness [26]. The Harrell concordance index (*C*-index) was measured to quantify the predictive performance of the nomograms [27]. The calibration curve, representing the agreement between the predicted and observed probabilities of relapse, was plotted to assess the calibration of the nomograms [28]. Decision curve analysis (DCA) was used to evaluate whether the nomograms were sufficiently robust for clinical practice. The net benefit was derived by calculating the difference between the true positive rate and the weighted false positive rate across different threshold probabilities [29]. To quantify the usefulness of the radiomics nomogram added by the radiomics signature, a net reclassification improvement (NRI) calculation was also applied [30].

### 2.6. Statistical Analysis

All statistical analyses were performed with R software (version 3.5.2, http://www.R-project.org) and X-tile software (version 3.6.1, Yale University School of Medicine, New Haven, Conn). LASSO Cox regression analysis was performed by using the “glmnet” package; we used ^“^nlambda = 100^”^ and ^“^maxit = 1000^”^ to calculate the signature; we used the “lambda.1se” to select critical features. Nomograms and calibration plots were performed with the “rms” package. The “hmisc” package was used for comparisons between *C*-indices. NRI was performed by using the “nricens” package. DCA was performed by using the “stdca” package. The reported statistical significance levels were all two-sided, with the statistical significance level set at 0.05.

## 3. Results

### 3.1. Clinical Characteristics

The patients were divided into two cohorts on the basis of hospitals: a training cohort and an independent validation cohort. The training cohort included 63 patients (36 men and 27 women; mean age, 24.10 ± 14.79 years; range, 5–71 years) from the first three hospitals. The external validation cohort consisted of 17 patients (13 men and 4 women; mean age, 31.12 ± 18.30 years; range, 14–64 years) from the other three hospitals. Thirty-three patients relapsed in one year, and 47 patients did not during the follow-up.

Clinical characteristics in the training and validation cohorts are summarized in Table 1. The clinical risk factors showed no significant differences between the relapse group and the nonrelapse group within the training and validation cohorts (*P* = 0.119–0.837) besides the HGB level in the training cohort (*P* = 0.018). For CT-assessed candidate characteristics, joint invasion showed significant differences within the two cohorts (*P* = 0.004 and 0.036, respectively). However, for CT-assessed morphology characteristics, solidity, sphericity, and irregularity had significant differences only for the training cohort (*P* = 0.003, 0.039, and 0.011, respectively).

The interobserver reproducibility of the extracted features was a perfect agreement with ICC > 0.80. Therefore, all segmentation results were based on the delineation of the first radiologist.

### 3.2. Construction of the Radiomics Signature

**Figure 2:**
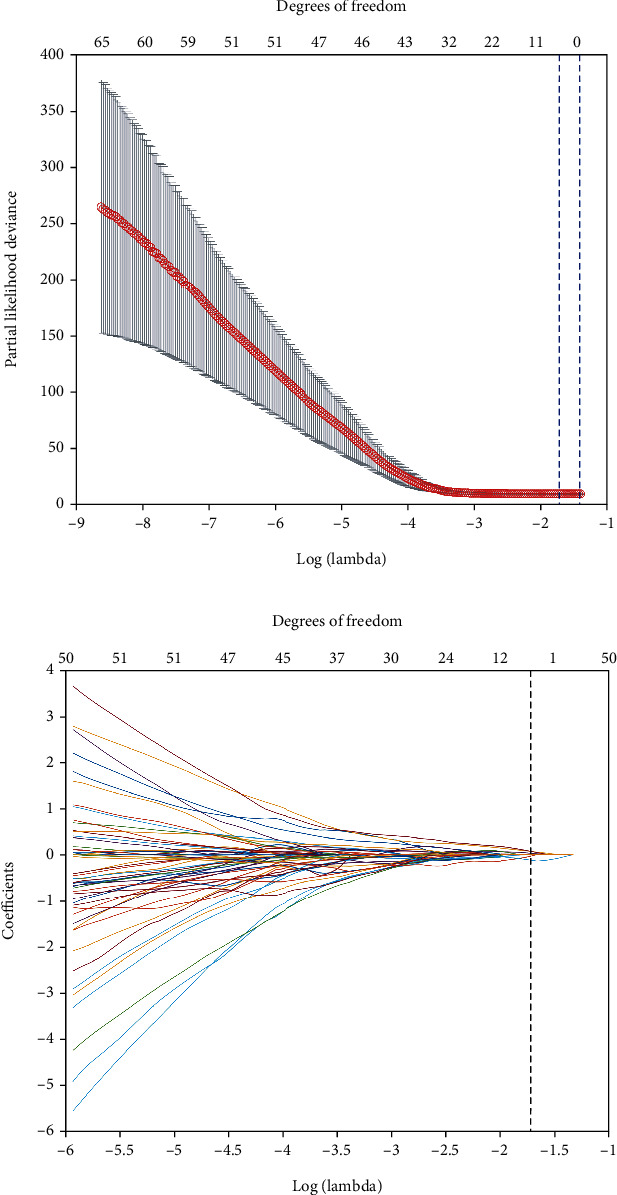
Texture feature selection using LASSO Cox regression model. (a) The partial likelihood deviation was plotted versus log (*λ*). The optimal parameter (*λ*) was selected using a sixfold cross-validation and 1 standard error rule. The optimal *λ* of 0.179 with log(*λ*) = −1.718 was selected. (b) LASSO coefficient profiles of 200 radiomics features were based on CT images. Finally, four features were selected, which are shown with black vertical dotted lines in the figure.

A total of 8750 features were extracted from each CT image. Among these features, 5675 reliable features with an ICC greater than 0.80 were selected for further analysis. After using the MRMR method, the top 200 prominently features remained. Of these top-ranked features, four features with nonzero coefficients in the LASSO Cox regression model were associated with relapse (Table 2). The radiomics signature was developed, based on the four selected features and their coefficients (Figure 2). The optimal value (the black vertical dotted lines) of the LASSO tuning parameter (*λ*) was 0.179. The radiomics signature was constructed, with a Rad-score calculated by using the following formula:

(1) $$\begin{array}{c} \text{Rad}‐\text{score}=\text{morph}\_\text{pca}\_\text{elongation}*0.2360+\text{HLL}\_64\_\text{Uniform}\_\text{stat}\_\mathrm{var}*0.6851+\text{LLL}\_64\_\text{Lloyd}\_\text{cm}\_\text{clust}\_\text{tend}*0.3164+\text{LHH}\_32\_\text{Lloyd}\_\text{szm}\_\text{glnu}*\left(-0.6421\right). \end{array}$$

**Figure 3:**
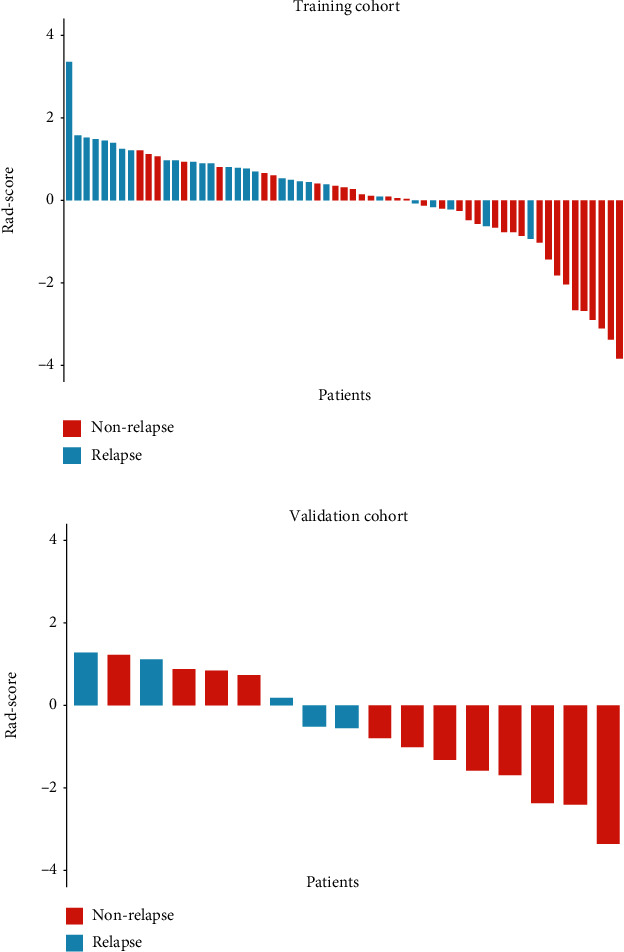
The radiomics score (Rad-score) for each patient in terms of the status of relapse for (a) training cohort and (b) validation cohort based on CT images. The red bars indicate nonrelapse patients, whereas the blue bars show relapse patients.

The distributions of the Rad-scores and state of relapse in the training and validation cohorts are shown in Figure 3. Patients in the relapse group generally had higher Rad-scores than nonrelapse patients.

### 3.3. Validation of the Radiomics Signature

The optimum cutoff between patients generated by the X-tile plot was −0.63 on the basis of the training cohort (Figure S2). Accordingly, patients were classified into the high-risk group (Rad − score ≥ −0.63) and low-risk group (Rad − score < −0.63). Relapse rates in the high-risk and low-risk groups of the training and validation cohorts are listed in Table 3.

**Figure 4:**
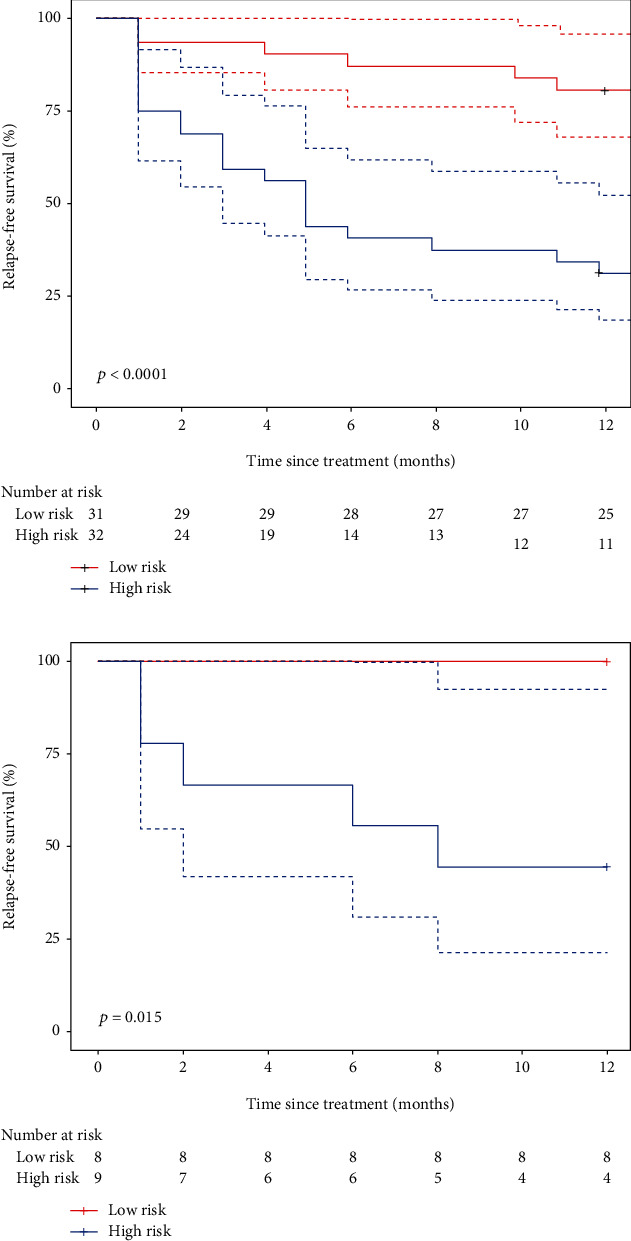
Kaplan-Meier survival curves according to the radiomics signature for patients with OS in (a) training cohort and (b) validation cohort. Dashed lines are two-sided CI of the survival curves (solid line).

The performances of the radiomics signature for relapse risk stratification determined by Kaplan-Meier curves are demonstrated in Figure 4. Kaplan-Meier curves showed a significant difference between the high-risk and the low-risk groups by using the G-rho rank test in both training and validation cohorts (*P* < 0.001 and *P* = 0.015, respectively). Patients with lower Rad-scores generally had the lower risk of relapse, although six (15.4%) patients confirmed with relapse were classified in the low-risk group in training and validation cohorts; high Rad-scores had the higher risk of relapse (66% and 56% patients with relapse were classified in the high-risk group).

### 3.4. Construction and Performance of the Radiomics Nomogram

**Figure 5:**
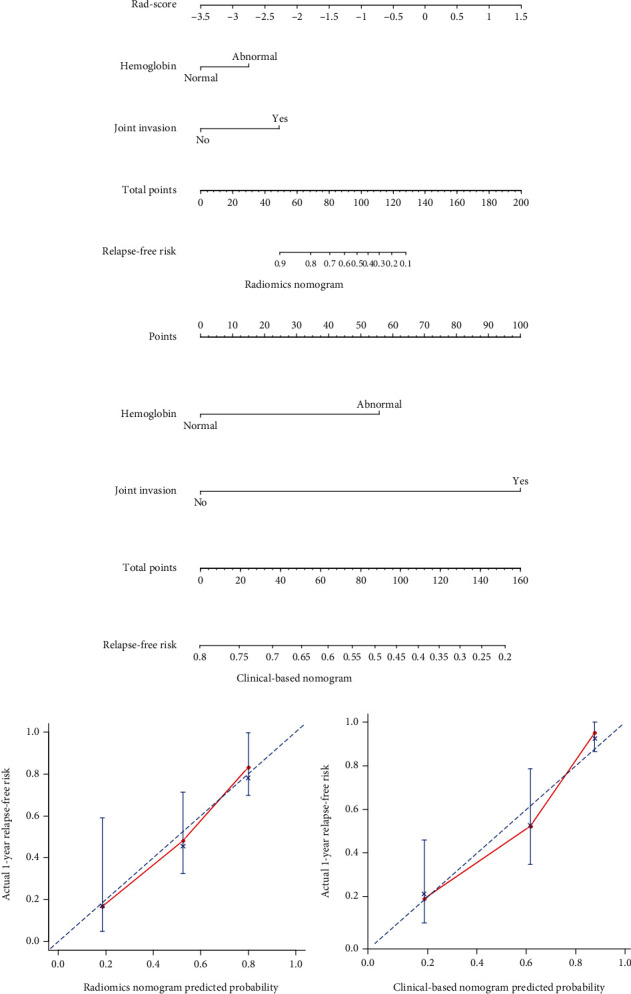
(a) Radiomics nomogram and (b) clinical-based nomogram. To use the nomogram, for each patient, locate the patient's Rad-score points on the Rad-score axis. Draw a line straight up to the point axis to calculate the points. Repeat the process for each of the risk factors and add the points up. Locate the final sum on the total point axis and draw a line straight down to find the patient's relapse-free risk. Calibration curves for (c) the radiomics nomogram and (d) the clinical-based nomogram show the calibration of each nomogram in terms of the agreement between the estimated and the observed relapse outcomes.

**Figure 6:**
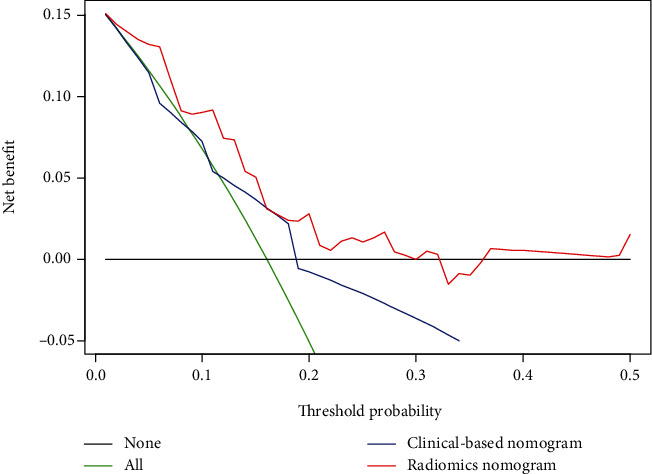
Decision curve analysis for the clinical-based nomogram and the radiomics nomogram. The *y*-axis represents the net benefit, whereas the *x*-axis represents the threshold probability.

A univariate Cox regression model was used to assess the predictive ability of clinical-based risk factors. HGB (HR: 2.3, 95% CI: 1.1–5) and joint invasion (HR: 3.9, 95% CI: 1.8–8.7) revealed significant predictive power (*P* = 0.025 and <0.001, respectively). The two factors were used to build the clinical-based nomogram and then integrated with the radiomics signature to construct the radiomics nomogram. The nomograms are presented in Figures 5(a) and 5(b). The calibration curves of the nomograms are shown in Figures 5(c) and 5(d); Radiomics calibration curve showed better agreement between the estimation with the nomogram and actual observation than the clinical-based curve. The discriminant performance of the radiomics signature improved when it was integrated with the radiomics nomogram along with the clinical-based risk factors. The *C*-index for the radiomics nomogram was 0.779 (95% CI: 0.70, 0.85) in the training cohort and 0.710 (95% CI: 0.53, 0.89) in the validation cohort. Compared with the clinical-based nomogram (*C*-index: 0.606; 95% CI: 0.51, 0.70 in the training cohort), the radiomics nomogram showed better discriminant capability (*P* < 0.001 for each comparison). Furthermore, the nomogram merged with signatures showed improved prediction accuracy for relapse outcome regarding NRI (0.507; 95% CI: −0.129–0.688, *P* < 0.001) compared with the clinical-based nomogram. A decision curve analysis showed that the radiomics nomogram had a higher overall net benefit than the clinical-based nomogram across the majority of the range of reasonable threshold probabilities (Figure 6).

## 4. Discussion

Due to the high heterogeneity of CT image acquisition in different institutions, there is widespread concern about the use of CT 3D image-based models for multicenter applications [31]. Therefore, based on multicenter data, we developed and externally validated a radiomics nomogram combining the radiomics signature and clinical-based risk factors for pretreatment relapse risk estimation.

In our study, tumor morphology including solidity, sphericity, and irregularity had a significant difference (*P* < 0.05) between patients with relapse and nonrelapse in the training cohort, which is consistent with previous studies that tumor morphology has been suggested as the risk factors for relapse in patients with some types of cancer including osteosarcoma by several studies [32, 33]. However, these morphological features have no statistical differences in the external validation cohort. We could not extract information regarding the response from other published series and immediately explain these results besides the small cohort size. More osteosarcoma patients should be enrolled to elucidate this biological phenomenon in the future.

We found HGB (HR: 2.3, 95% CI: 1.1–5) and joint invasion (HR: 3.9, 95% CI: 1.8–8.7) to be useful for prediction of relapse risk in osteosarcoma patients (*P* = 0.025 and <0.001, respectively); thus, HGB and joint invasion were used to build the clinical nomogram and integrate them with the Rad-score to construct the radiomics nomogram. But neither of them could be a single factor to predict relapse accurately because even if the clinical risk factors to be used were available, a great deviation would be generated by using only one clinical risk factor for prognosis in the imbalance cohort [34]. Although, previous studies have shown that clinical risk factors are independent prognostic factors for the survival of patients with osteosarcoma [35].

In our study, the radiomics signature demonstrated that about 43% of patients (Rad − score ≥ –0.63) were predicted by the signature to relapse within a year. It is noteworthy that variance, cluster tendency, and gray level nonuniformity (GLN) were observed to be significantly correlated with relapse in the combined whole dataset. Cluster tendency, the measure of voxel clusters of similar gray level values, has been confirmed to play critical roles in the survival prediction and stratification of cancer patients [36]. Difference variance is a measure of heterogeneity that places higher weights on differing intensity level pairs that deviate more from the mean. GLN measures the variability of gray level intensity values in the image, with a higher value indicating more heterogeneity in intensity values. Our results confirmed this concept that radiomics has emerged as a potential solution to predict relapse for osteosarcoma patients [37].

In recent studies, Lin et al. [17] and Wu et al. [14] have, respectively, developed a CT-based radiomics nomogram for evaluation of curative effect and prognosis in osteosarcoma, which presented an AUC of 0.840 and 0.843 in the validation cohort, respectively. Although they had more numbers of patients in their study and the performance of their radiomics model was superior to our research, they did not have external validation, and *they only analyzed the patients with high-grade osteosarcoma while we studied all-grade osteosarcoma patients*. Furthermore, we included patients from six different institutions and from different CT devices, while the same CT scanner was selected in their study. Different CT image acquisitions result in the difference of radiomics features [38, 39], which might lead to bias and could explain the poor performance of the radiomics model we developed. Nevertheless, our study based on multicenter data may make our results more incremental value than single-center data trials. Without adjusting any key features and their corresponding weights, a radiomics signature can be built, so the application of the radiomics nomogram was very straightforward. The calibration plots demonstrated excellent agreement between risk stratification and nomogram prediction. Furthermore, the integration of the clinical-based risk factor with the nomogram showed improved prediction accuracy for risk outcome regarding NRI (0.507; 95% CI: −0.129–0.688, *P* < 0.001) for individualized early relapse prediction comparing with the nomogram without it. A decision curve analysis further confirmed the potential value of this radiomics-based nomogram that within most reasonable threshold probabilities, the radiomics nomogram had a greater overall net benefit than the clinical nomogram.

There were several limitations to the current study. First, this study included a relatively small sample size. One reason was that the incidence rate of osteosarcoma is very low and it is very difficult to collect patient data. Another reason was that one reason was that the incidence rate of osteosarcoma is very low and it is very difficult to collect patient data. Another reason was that the indications for surgery may have differed among the participating hospitals, and this could have resulted in a selection bias. Second, genetic research was not included in our study. Previous studies have found a series of metastasis-related genes in osteosarcoma [40]. Third, the relapse potential may be related to the location of osteosarcoma tumors. In future work, we will develop a series of prediction models for different locations of tumors. At last, the standard of care for pretreatment imaging in patients with osteosarcoma is MRI, not CT, in some hospitals limiting the clinical implication of our results.

In summary, this externally verified radiomics nomogram that incorporated both the radiomics signature and clinical-based risk factors can increase the predictive value of the osteosarcoma patient's relapse risk, which supports the clinical application in different institutions.

## Figures and Tables

**Table 1:** Clinical risk factors and CT-assessed candidate predictors of the patients.

Characteristic	Training cohort			Validation cohort		
	Relapse	Nonrelapse	*P*	Relapse	Nonrelapse	*P*
Clinical risk factors						
Patients	26 (41.3%)	37 (58.7%)		5 (29.4%)	12 (70.6%)	0.271^a^
Sex (no. [%])			0.941^a^			0.825^a^
Male	15 (57.7%)	21 (56.8%)		4 (80.0%)	9 (75.0%)	
Female	11 (42.3%)	16 (43.2%)		1 (20.0%)	3 (25.0%)	
Age (years)			0.905^a^	32.60 ± 20.80	30.50 ± 18.11	0.793^a^
<30	24 (80.0%)	26 (78.8%)		3 (60.0%)	8 (66.7%)	
≥30	6 (20.0%)	7 (21.2%)		2 (40.0%)	4 (33.3%)	
Tumor location			0.253^a^			0.119^a^
Long bone	17 (65.4%)	29 (78.4%)		3 (60.0%)	11 (91.7%)	
Flat bone	9 (34.6%)	8 (21.6%)		2 (40.0%)	1 (8.3%)	
ALP (alkaline phosphatase) level (IU/L)			0.966^a^			0.169^a^
Normal	17 (65.4%)	24 (64.9%)		2 (40.0%)	9 (75.0%)	
Abnormal	9 (34.6%)	13 (35.1%)		3 (60.0%)	3 (25.0%)	
Hemoglobin level (mmol/L)			0.031^a^			0.149^a^
Normal	12 (46.2%)	27 (73.0%)		1 (20.0%)	7 (58.3%)	
Abnormal	14 (53.8%)	10 (27.0%)		4 (80.0%)	5 (41.7%)	
CT-assessed candidate predictors						
Tumor volume (mm^3^)	281090.14 ± 306387.32	295712.47 ± 307695.10	0.856^a^	1673453.67 ± 2236478.54	602010.56 ± 799571.06	0.300^a^
Surface	105.76 ± 35.39	134.46 ± 85.98	0.105^a^	377.23 ± 434.90	268.12 ± 272.45	0.493^a^
Size	0.83 ± 0.66	0.82 ± 0.96	0.765^a^	0.86 ± 0.03	0.86 ± 0.07	0.978^a^
Solidity	0.69 ± 0.10	0.80 ± 0.16	0.003^a^	0.80 ± 0.20	0.80 ± 0.18	0.947^a^
Eccentricity	251.94 ± 180.57	317.44 ± 265.96	0.275^a^	1169.81 ± 148.00	522.12 ± 578.42	0.343^a^
Compactness	3.37 ± 2.04	0.9 ± 1.66	0.570^a^	6.21 ± 4.78	4.43 ± 3.71	0.371^a^
Sphericity	0.78 ± .0.17	0.68 ± .0.19	0.039^a^	0.72 ± 0.25	0.77 ± 0.29	0.726^a^
Support vector regression	1.20 ± 0.43	1.42 ± 0.74	0.169^a^	0.98 ± 0.41	1.11 ± 0.39	0.529^a^
Irregularity	1.33 ± 0.26	1.56 ± 0.40	0.011^a^	1.55 ± 0.62	1.53 ± 0.69	0.941^a^
Joint invasion			0.002^a^			0.036^a^
Yes	17 (65.4%)	10 (27.0%)		4 (80.0%)	3 (25.0%)	
No	9 (34.6%)	27 (73.0%)		1 (20.0%)	9 (75.0%)	
Pathologic fracture			0.184^a^			0.253^a^
Yes	4 (15.4%)	2 (5.4%)		0 (0.0%)	0 (0.0%)	
No	22 (84.6%)	35 (94.6%)		5 (100.0%)	12 (100.0%)	

^∗^ *P* value is less than 0.05. ^a^Chi-square test.

**Table 2:** Radiomics feature selection results.

Feature family	Feature name	Tag	Coefficients
Morphology	Elongation	morph_pca_elongation	0.2360
Wavelet-based statistics	Variance	HLL_64_uniform_stat_var	0.6851
Wavelet-based gray level cooccurrence matrix	Cluster tendency	LLL_64_lloyd_cm_clust_tend	0.3164
Wavelet-based gray level size zone matrix	Gray level nonuniformity (GLN)	LHH_32_lloyd_szm_glnu	-0.6421

**Table 3:** Relapse rate in high-risk and low-risk groups.

Parameter	Training cohort		Validation cohort	
	High-risk group	Low-risk group	High-risk group	Low-risk group
No. of patients	32	31	9	8
No. of patients with relapse	21	6	5	0
Rate of patients with relapse	66%	19%	56%	0%

## Data Availability

The data used to support the findings of this study are available from the corresponding author upon request.

## Supplementary Materials

Supplementary Materials Supplementary 1: The Criteria for Inclusion and Exclusion. Supplementary 2: Treatments. Supplementary 3: Follow-up Details. Supplementary 4: The Criteria of Serum Markers. Supplementary 5: Feature Extraction Methodology. Supplementary 6: Feature Selection Procedure. Supplementary Table S1: CT Image Acquisition Parameters. Supplementary Figure S1: The Recruitment Pathway for Patients in This Study. Supplementary Figure S2: X-tile Plot of the Radiomics Signature in the Training Cohort.
