# Supplementary material for: Pretreatment Prediction of Relapse Risk in Patients with Osteosarcoma Using Radiomics Nomogram Based on CT: A Retrospective Multicenter Study
Source: Biomed Res Int. 2021 Feb 4;2021:6674471. doi: 10.1155/2021/6674471 (PMC7878076; doi:10.1155/2021/6674471)
Supplement: Supplementary Materials — Supplementary 1: The Criteria for Inclusion and Exclusion. Supplementary 2: Treatments. Supplementary 3: Follow-up Details. Supplementary 4: The Criteria of Serum Markers. Supplementary 5: Feature Extraction Methodology. Supplementary 6: Feature Selection Procedure. Supplementary Table S1: CT Image Acquisition Parameters. Supplementary Figure S1: The Recruitment Pathway for Patients in This Study. Supplementary Figure S2: X-tile Plot of the Radiomics Signature in the Training Cohort. [file 6674471.f1.docx]

**Pretreatment** **Prediction of Relapse Risk in Patients with Osteosarcoma using Radiomics Nomogram Based on CT: a Retrospective Multicenter Study**

**Supplementary Material**

1. **The Criteria for Inclusion and Exclusion**
2. **Treatments**
3. **Follow-up Details**
4. **The Criteria of Serum Markers**
5. **Feature Extraction Methodology**
6. **Feature Selection Procedure**
7. **Supplementary Table and Figures**

**7.1. CT Image Acquisition Parameters**

**7.2 Figure S1. The Recruitment Pathway for Patients in This Study**

**7.3 Figure S2. X-tile Plot of the Radiomics Signature in the Training Cohort**

1. **The Criteria for Inclusion and Exclusion**

*The Inclusion Criteria were as Follows*

(a) patients with osteosarcoma diagnosed by multidisciplinary teams; (b) an open biopsy or CT-guided core needle biopsy, pathologically evaluated by specialized sarcoma pathologists; (c) surgery was performed after the completion of neoadjuvant chemotherapy; (d) a standard CT scan performed at the time of diagnosis; (e) clinical characteristics available.

*The Exclusion Criteria were as Follows*

(a) patients underwent treatment (chemotherapy or surgery resection) before undergoing a CT scan; (b) the patient underwent biopsy in other external hospitals and pretreatment pathological results were not available; (c) the patient performed pretreatment CT imaging in other external hospitals and CT data was missed; (d) patients suffered from other synchronous cancers; (e) clinical information was unavailable; (f) death by a cause other than osteosarcoma; (h) The follow-up of patients was within one year.

1. **Treatments**

The treatment protocols all followed the National Comprehensive Cancer Network (NCCN) guidelines and be updated timely in the different hospitals [1].

*Neoadjuvant Chemotherapy*

The chemotherapy regimen was the combination of the AP-M-I protocol (doxorubicin, cisplatin-methotrexate-ifosfamide) with 1-2 cycles prior to surgery and 3-4 cycles post-operatively. Chemotherapy drugs were as follows: Adriamycin (ADM), cisplatin (CDP), bleomycin (BLM), cyclophosphamide (CTX) or ifosfamide (IFO), actinomycin (ACD), and high-dose methotrexate (MTX).

*Main Indications for Limb Salvage Surgery*

(a) Enneking stage I A, I B and IIA patients, and some stage IIB chemotherapy-sensitive patients; the major blood vessels and nerves are not affected; (b) if the local soft-tissue condition permit, the extensive resection can be achieved; (c) there is no metastatic lesion or the metastatic lesion can be cured; (d) the general condition is good, and the patient has a strong desire for limb salvage.

*Contraindications for Limb Salvage Surgery*

When the recurrent tumors have huge tumor bodies, poor differentiation, and bad soft-tissue condition, or when the major blood vessels and nerves surrounding the tumor are invaded by the tumor, it is advisable to perform amputation surgery.

1. **Follow-up Details**

Routine laboratory tests and plain radiography has been used routinely for these patients every month during postoperative chemotherapy and every 3 months after postoperative chemotherapy in the first year, Additional CT was routinely performed if lung metastasis was suspected. Enhanced MRI scans of the surgical location were obtained in case of clinical or radiological suspicion of the development of local relapse. If other abnormal findings were detected on follow-up evaluations, CT, MRI, and ultrasound were conducted. Histological examination was additionally performed to verify relapse when local relapse was not excluded by imaging modalities. The longest follow-up time was 12 months and the shortest follow-up time was 1 month.

1. **The Criteria of Serum Markers**

The serum alkaline phosphatase (ALP) levels were measured in international units (IU), and the activity of ALP was estimated by the p-nitrophenyl phosphate method. ALP ranges of 60.0-300.0 IU/L for patients ≤14 years and 38.0-115.5 IU/L for patients > 15 years were considered normal [2]. The threshold values for serum HGB levels were defined as: low HGB was defined as levels b 7.3 mmol/l for females and b 8.3 mmol/l for males [3].

1. **Feature Extraction Methodology**

Feature extraction was conducted from the ROI of CT image by using in-house feature extraction software with algorithms implemented in MATLAB 2015b (Math-works, Natick, MA, USA). The extracted features conform to the definitions in Image Biomarker Standardization Initiative (IBSI) [4]. These features can be divided into 7 different families: (a) 29 morphological features; (b) 2 local intensity features; (c) 18 intensity-based statistical features; (d) 23 intensity histogram features; (e) 7 intensity-volume histogram features; (f) 408 texture features which derived from gray level co-occurrence matrix (GLCM), gray level run length matrix (GLRLM), gray level size zone matrix (GLSZM), gray level distance zone matrix (GLDZM), neighborhood gray tone difference matrix (NGTDM), neighborhood grey level dependence matrix (NGLDM); (g) wavelet features: the discrete undecimated 3D wavelet transform is used in this study. The original image was decomposed into 8 decompositions for every patient. The decomposed images from $X$ are marked as $X\_LLL$, $X\_LLH$, $X\_LHL$, $X\_LHH$, $X\_HLL$, $X\_HLH$, $X\_HHL$ and $X\_HHH$. Then texture features are extracted from each decomposition. In this study “Coiflet 1" wavelet was applied to the original CT images.

The parameter configurations used in this study are as follows: (a) CT images and their morphological masks were resampled to 2D and 3D isotropic voxel spacing (voxel size: 1mm and 2mm, respectively) with trilinear interpolation algorithm; (b) For calculation texture features, CT images were discretized by fixed bin number (bin numbers used in this study: 16, 32, 64, 128) with two quantization method (uniform quantization and Max-Lloyd quantization) [5].

1. **Feature Selection Procedure**

Features were normalized using the Z-score method to perform the subsequent screening. Feature selection was performed in 2 steps to select the optimal signatures via the training cohort.

Firstly, we used the Minimum Redundancy Maximum Relevance (MRMR) [6] to evaluate the reliance on features with the labels and penalize redundancy between features. The scoring criterion is a weighted sum of feature relevance and redundancy and is given by

$$J_{mrmr}(X_{k})=I(X_{k};Y)-\frac{1}{\left|S\right|}\sum_{X_{j}\in S} I(X_{k};X_{j})$$

Here the first term $I(X_{k};Y)$is the mutual information between the feature$X_{k}$ and class labels, which indicates the feature relevancy. The second term $\sum_{X_{j}\in S} I(X_{k};X_{j})$corresponds to the feature redundancy. So, a feature is only going to get the high score if it is highly relevant to the class labels and also non-redundant to the set of already selected feature set$S$. By using MRMR analysis, the top 200 features remained.

Secondly, the least absolute shrinkage and selection operator (LASSO) based Cox regression algorithm, which is applicable for high-dimensional data reduction [7], was performed to select the most important predictive features. LASSO algorithm reduced the coefficients of relapse-unrelated variables to zero; variables with non-zero coefficients were retained. To select optimal variables in LASSO Cox regression, we performed 50 iterations of 6-fold cross-validation with partial-likelihood deviance minimization criteria [8] from the training cohort by ‘glmnet’ package. The model with the minimum deviance as selected. In this study, four features with non-zero coefficient were selected via LASSO.

1. **Supplementary Table and Figures**
   1. **Table S1. CT Image Acquisition Parameters**

| **Table S1. CT Image Acquisition Parameters** | | | | | | | | |
| --- | --- | --- | --- | --- | --- | --- | --- | --- |
|  | **Scanner** | **Tube voltage** | **Tube current** | **Rotation time** | **Detector collimation** | **Slice thickness** | **Field of view** | **Pixel matrix** |
| TAH-SMU | Toshiba Aquilion 64-slice CT scanner | 120 kV | 220 mA | 0.5s | 42 × 5 mm | 5.0 mm | 300 × 300 mm^2^ | 512 × 512 pixels |
| GPPH | GE LightSpeed VCT 64-slice CT scanner | 120 kV | 160 mA | 0.5s | 38× 3 mm | 5.0 mm | 300 × 300 mm^2^ | 512 × 512 pixels |
| ZH-SMU | Philips 256-slice Brilliance iCT system | 120 kV | 140 mA | 0.5s | 46× 5 mm | 3.0 mm | 300 × 300 mm^2^ | 512 × 512 pixels |
| FAH-GMU | Siemens 128-slice Somatom Definition AS+ CT scanner | 120 kV | 80 mA | 0.5s | 128× 0.6 mm | 4.0 mm | 300 × 300 mm^2^ | 512 × 512 pixels |
| FAH-SYU | Siemens Somatom Sensation 16-detector CT scanner | 120 kV | 200 mA | 0.5s | 16 × 0.75 mm | 5.0 mm | 300 × 300 mm2 | 512 × 512 pixels |
| TAH-SYU | Toshiba Aquilion 64-slice CT scanner | 120 kV | 250 mA | 0.5s | 64 × 0.5 mm | 3.0 mm | 300 × 300 mm^2^ | 512 × 512 pixels |
| Abbreviations: *TAH-SMU*, Third Affiliated Hospital of Southern Medical University; *GPPH*, Guangdong Provincial People's Hospital;  *ZH-SMU*, Zhujiang Hospital of Southern Medical University; *FAH-GMU*, The First Affiliated Hospital of Guangzhou Medical University; *FAH-SYU*, the Third Affiliated Hospital of Sun Yat-sen University*；TAH-SYU*, the Third Affiliated Hospital of Sun Yat-sen University. | | | | | | | | |

**7.2 Figure S1. The Recruitment Pathway for Patients in This Study**


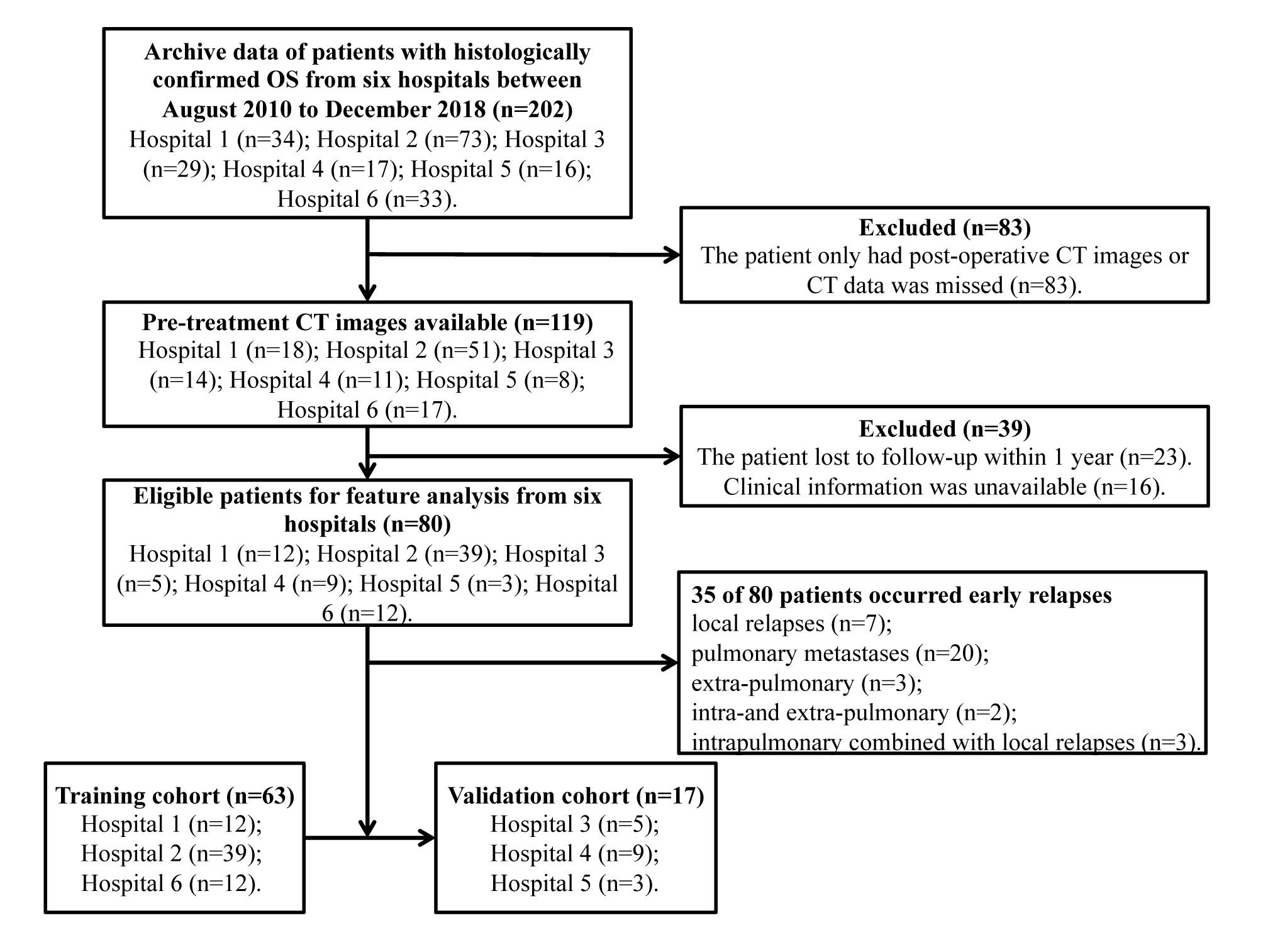


**Fig. S1.** **The recruitment pathway for patients in this study.**

**7.3 Figure S2. X-tile Plot of the Radiomics Signature in the Training Cohort**


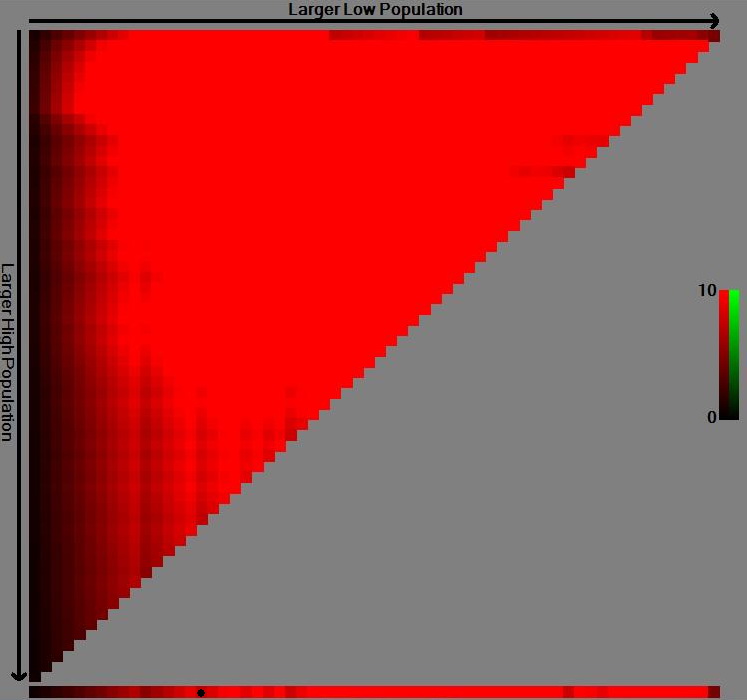


**Fig. S2.** **X-tile plot of the radiomics signature in the training cohort.** The colors in the plot represent the strength of the association at each division, ranging from low (black) to high (bright red or green). Red represents the inverse association between the Rad-score and Relapse. The x-axis represents all potential cutoff points, from low to high (left to right), that define a low subset, whereas the y-axis represents cutoff points from high to low (top to bottom) that define a high subset. The optimum cut point is highlighted by the black dot on the x-axis.

198 x 185 mm (96 x 96 DPI)

**REFERENCE**

[1] J. S. Biermann, W. Chow, D. R. Reed, D. Lucas, D. R. Adkins, M. Agulnik, R. S. Benjamin, B. Brigman, G. T. Budd and W. T. Curry, "NCCN guidelines insights: bone cancer, version 2.2017," *Journal of the National Comprehensive Cancer Network*, vol. 15, no. 2, pp. 155-167, 2017.

[2] M. S. Kim, S. Y. Lee, T. R. Lee, W. H. Cho, W. S. Song, J. S. Koh, J. A. Lee, J. Y. Yoo and D. G. Jeon, "Prognostic nomogram for predicting the 5-year probability of developing metastasis after neo-adjuvant chemotherapy and definitive surgery for AJCC stage II extremity osteosarcoma," *Ann Oncol*, vol. 20, no. 5, pp. 955-960, 2009.

[3] N. Aggerholm-Pedersen, K. Maretty-Kongstad, J. Keller, S. Baerentzen and A. Safwat, "The Prognostic Value of Serum Biomarkers in Localized Bone Sarcoma," *Transl Oncol*, vol. 9, no. 4, pp. 322-328, 2016.

[4] A. Zwanenburg, S. Leger, M. Vallières and S. Löck, "Image biomarker standardisation initiative - feature definitions," 2016.

[5] M. Vallieres, C. R. Freeman, S. R. Skamene and I. El Naqa, "A radiomics model from joint FDG-PET and MRI texture features for the prediction of lung metastases in soft-tissue sarcomas of the extremities," *Phys Med Biol*, vol. 60, no. 14, pp. 5471-5496, 2015.

[6] H. Peng, F. Long and C. Ding, "Feature selection based on mutual information: criteria of max-dependency, max-relevance, and min-redundancy," *IEEE Trans Pattern Anal Mach Intell*, vol. 27, no. 8, pp. 1226-1238, 2005.

[7] R. Tibshirani, "The lasso method for variable selection in the Cox model," *Stat Med*, vol. 16, no. 4, pp. 385-395, 1997.

[8] C. B. Roosen and T. J. Hastie, "Logistic response projection pursuit," *AT&T Bell Laboratories, Doc. BL011214-930806-09TM, Murray Hill, NJ*, 1993.
